# CGG repeat expansions in Charcot-Marie-Tooth disease: insights from the 100 000 Genomes Project

**DOI:** 10.1136/jnnp-2025-336590

**Published:** 2025-07-11

**Authors:** Alessandro Bertini, Stefano Facchini, Ilaria Quartesan, Riccardo Currò, Ricardo Parolin Schnekenberg, Natalia Dominik, Gustavo Alves, Lucia Ferullo, Arianna Tucci, Henry Houlden, Mary M Reilly, Andrea Cortese

**Affiliations:** 1 Department of Neuromuscular Diseases, UCL Queen Square Institute of Neurology, London, UK; 2 Unit of Rare Neurological Diseases, Department of Clinical Neurosciences, Fondazione IRCCS Istituto Neurologico Carlo Besta, Milan, Italy; 3 Department of Brain and Behavioral Sciences, University of Pavia, Pavia, Italy; 4 Clinical Hospital of Ribeirão Preto, Department of Neurosciences and Behaviour Sciences, University of São Paulo, Ribeirão Preto, Brazil; 5 Department of Clinical and Experimental Sciences, University of Brescia, Brescia, Italy; 6 William Harvey Research Institute, Faculty of Medicine and Dentistry, Queen Mary University of London, London, UK

**Keywords:** NEUROGENETICS, NEUROPATHY, NEUROMUSCULAR

## Abstract

**Background:**

CGG expansions in *NOTCH2NLC* and *LRP12* were recently identified as a cause of Charcot-Marie-Tooth disease (CMT) in 1.2%–10.6% of genetically undiagnosed patients in China, Taiwan and Japan. However, their relevance in CMT patients of different ethnic origin is still unknown.

**Methods:**

Here, we leveraged short-read whole genome sequencing data from the 100 000 Genomes Project to investigate the presence and frequency of CGG expansions in *NOTCH2NLC*, *LRP12* and additional genes associated with oculopharyngodistal myopathy (OPDM), in 560 genetically unsolved patients diagnosed with CMT and 32 509 non-neurological controls.

**Results:**

Repeat expansions in *NOTCH2NLC*, *LRP12*, *RILPL1*, *NUTM2B-AS1* and *ABCD3* were absent from 560 genetically unsolved patients with CMT, mostly of Northern European ancestry. One patient of African ancestry carried an expanded allele in *GIPC1*, below the reported pathogenic threshold. However, rare expansions in this gene, as well as in *NOTCH2NLC*, *NUTM2B-AS1* and *ABCD3*, were also detected in controls (≤0.05%). The distribution of repeat size at these loci varied significantly across different ethnicities, with larger non-pathogenic intermediate alleles of *NOTCH2NLC* and *LRP12* typically observed in East Asians.

**Conclusions:**

CGG expansions in *NOTCH2NLC*, *LRP12* and other OPDM-associated genes do not appear to be a relevant cause of CMT in the UK. The larger size of non-pathogenic intermediate alleles of *NOTCH2NLC* and *LRP12* in East Asians could explain their ancestry-specific propensity to further expand into the full pathogenic range.

## Introduction

 CGG repeat expansions in the human genome cause a broad range of neurological conditions, from intellectual disability to neuronal intranuclear inclusion disease (NIID), dementia, parkinsonism, ataxia and neuromuscular disease.^1^ More specifically, expansion in six functionally unrelated genes*—NOTCH2NLC*,^2 3^ *NUTM2B-AS1*,^2^ *LRP12*,^2^ *GIPC1*,^4^ *RILPL1*^5^ and *ABCD3*^6^—has been associated with a specific type of muscle disease leading to progressive facial, pharyngeal and distal limb weakness and termed oculopharyngodistal myopathy (OPDM), which, although rare, appears to be relatively more frequent in East Asians.^1^

More recently, CGG expansions in *NOTCH2NLC* and *LRP12* were also identified as a common cause of inherited peripheral neuropathy in individuals from China, Taiwan and Japan, accounting for 1.2%–10.6% of Charcot-Marie-Tooth (CMT) and distal hereditary motor neuropathy cases (dHMN).^7^,^11^ Moreover, up to 47% of *NOTCH2NLC*-NIID patients show a pure small fibre neuropathy, while subclinical large-fibre involvement is described in 53%–96% of cases.^12 13^ These findings suggest that CGG expansion in these genes may be a relevant cause of inherited neuropathy in the East-Asian population.

As over 50% of axonal CMT individuals remains unsolved across different series, including in a recent cohort of patients from the UK who underwent short-read whole genome sequencing (srWGS),^14^ we raise the question of whether CGG expansion in one or more of these genes should be screened in all CMT cases, independently of their ancestry.

In this study, we leveraged a large collection of srWGS data from the 100 000 Genomes Project (100K GP)^15^ (genomicsengland.co.uk/initiatives/100000-genomes-project) to investigate the presence and frequency of CGG expansions in *NOTCH2NLC* and *LRP12*, as well as other OPDM-associated genes, in genetically unsolved patients with CMT from the UK, and to assess the normal and pathological variation of these microsatellites in ethnically diverse populations.

## Methods

We used Expansion Hunter V.5 to profile the CGG repeat size in *NOTCH2NLC*, *LRP12*, *GIPC1*, *RILPL1*, *NUTM2B-AS1* and *ABCD3* loci from short-read WGS data in 560 probands with genetically unsolved CMT (495 Europeans, 30 South Asians, 19 Africans, 15 Americans, 1 East Asian) and 32 509 non-neurological controls (27 392 Europeans, 2387 South Asians, 1306 Africans, 1148 Americans and 276 East Asians).

We considered fully expanded alleles >50 repeats, considering that repeats larger than 50 units are typically underestimated by srWGS,^16^ and the reported pathogenic threshold for all these loci falls above this repeat size. We previously used this approach to identify *ABCD3* CGG expansions as a cause of OPDM,^6^ demonstrating that, although large expansions cannot be accurately sized, the method remains effective for identifying outliers that deviate significantly from the intermediate, non-pathogenic repeat size range.

Distribution of CGG repeat number was expressed as median, Q1 (first quartile)–Q3 (third quartile), and 99th/99.9th percentiles. Mann-Whitney U test/Kruskal-Wallis test and two-tailed χ^2^ test were used to compare distribution of CGG repeats and the frequency of fully expanded alleles across groups, as appropriate. Throughout the statistical analysis, the significance level was set at 0.05 (significant: <0.05).

## Results

**Figure 1:**
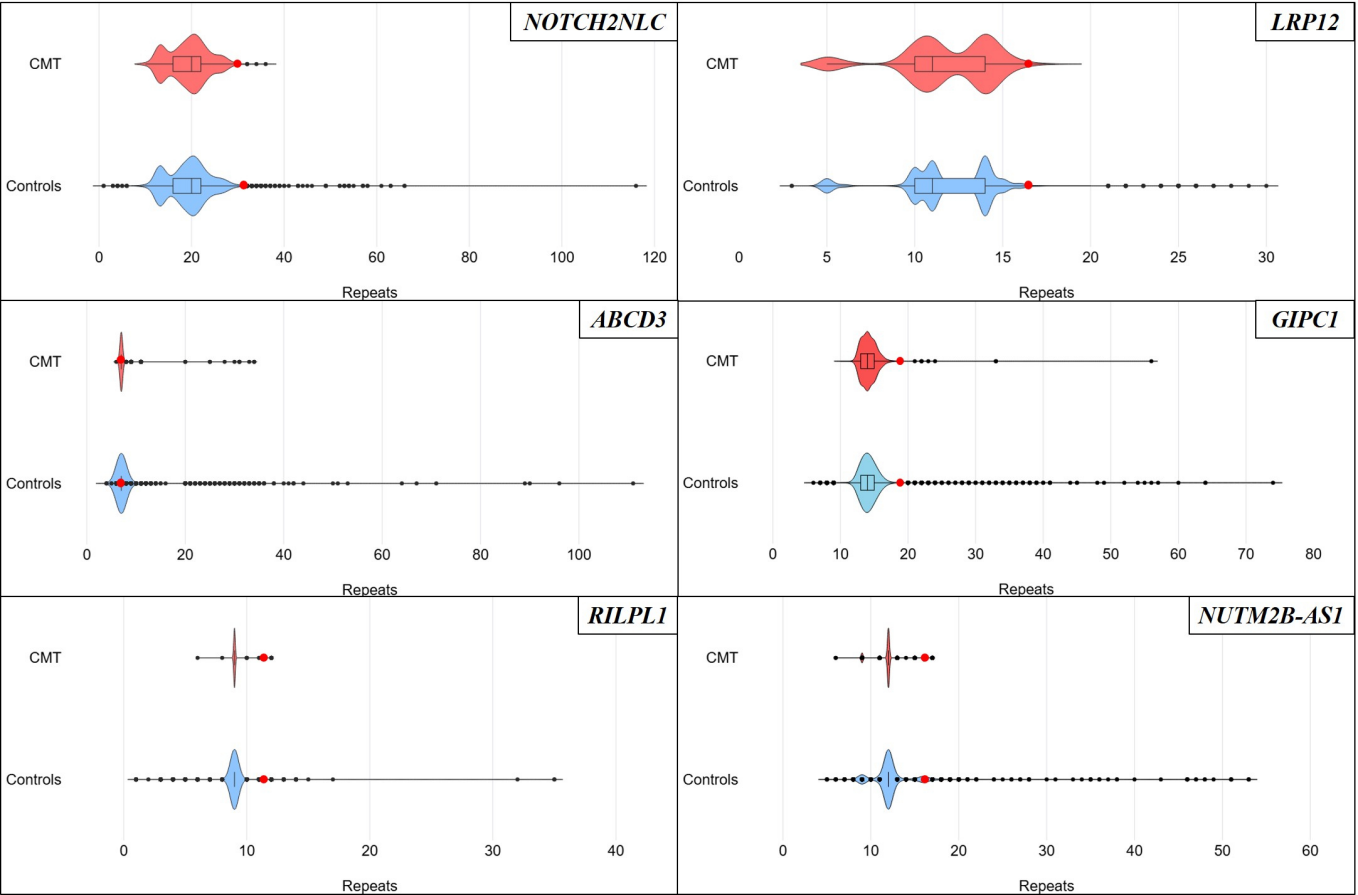
Repeat size distribution in CMT patients and non-neurological controls from the 100 000 Genomes Project cohort. n=1120 alleles; Controls=non-neurological controls, n=65 018 alleles. The box plots highlight the IQR and median. Red dots indicate the 99th percentile. CMT, Charcot-Marie-Tooth.

Repeat expansions >50 units in *NOTCH2NLC*, *LRP12*, *RILPL1*, *NUTM2B-AS1* and *ABCD3* were absent from 560 genetically unsolved individuals with CMT. An expanded allele in *GIPC1* corresponding to 56 CGG repeats, below the reported pathogenic threshold of 76 repeats,^1^ was found in one patient with demyelinating CMT of African ancestry (figure 1; online supplemental table 1).

Expanded alleles in *NOTCH2NLC*, *GIPC1*, *NUTM2B-AS1* and *ABCD3* were present, although very rare (allele frequency ≤0.05%), in controls, while no expansion was found in *LRP12* and *RILPL1*.

Because of the known technical limitation of srWGS in sizing large repeat expansion, we were not able to accurately estimate the repeat size of the expanded alleles in controls.

The distribution of CGG repeat size was similar between CMT patients and controls for all considered genes. Conversely, we observed a statistically significant variation depending on their ethnicity (p=0.011 for *ABCD3*; p<0.001 for other genes). When considering the upper tail of the repeat size distribution, specifically, the 99th percentile (or 99.9th percentile for *NUTM2B-AS1*), non-pathogenic intermediate alleles showed distinct population differences. Intermediate alleles in *NOTCH2NLC, LRP12, NUTM2B-AS1* and *RILPL1* were larger in East Asians, while in *GIPC1,* they were larger in both East Asians and Africans. In contrast, *ABCD3* appeared larger in Europeans (table 1; online supplemental figure 1). Frequency of intermediate expansion of *NOTCH2NLC* was evenly distributed across all age groups (0–30, 31–60, >60 years), limiting a possible bias towards identification of these expansions in younger, and possibly still presymptomatic, individuals.

**Table 1:** Repeat size distribution and frequency of expanded alleles across non-neurological controls of different ancestry

	EUR n=27 392	SAS n=2387	AFR n=1306	AMR n=1148	EAS n=276	P value*
*NOTCH2NLC*						
Repeat size, median (Q1–Q3)	20 (16–22)	19 (15–21)	20 (17–22)	20 (16–22)	20 (15–23)	**<0.001**
Repeat size (99° percentile)	29	31	30	29	**35**	
Frequency of allele with >50 repeats	0.0005 (29/54784)	0.0006 (3/4774)	0.0008 (2/2612)	0.0013 (3/2296)	**0.0018** (**1/548**)	**0.048**
*LRP12*						
Repeat size, median (Q1–Q3)	11 (10–14)	11 (10–14)	10 (9–12)	11 (10–14)	11 (11–14)	**<0.001**
Repeat size (99° percentile)	16	17	18	17	**19**	
Frequency of allele with >50 repeats	0 (0/54784)	0 (0/4774)	0 (0/2612)	0 (0/2296)	0 (0/548)	/
*ABCD3*						
Repeat size, median (Q1–Q3)	7 (7-7)	7 (7-7)	7 (7-7)	7 (7-7)	7 (7-7)	**0.011**
Repeat size (99° percentile)	**12**	9	11	7	7	
Frequency of allele with >50 repeats	0.0002 (10/54784)	0 (0/4774)	0 (0/2612)	0.0004 (1/2296)	0 (0/548)	0.124
*GIPC1*						
Repeat size, median (Q1–Q3)	14 (13–15)	14 (13–14)	14 (13–15)	14 (13–15)	14 (14–15)	**<0.001**
Repeat size (99° percentile)	18	24	32	23	**34**	
Frequency of allele with >50 repeats	0.0002 (11/54784)	0.0006 (3/4774)	0.0034 (9/2612)	0.0021 (5/2296)	**0.0036** (**2/548**)	**<0.001**
*RILPL1*						
Repeat size, median (Q1–Q3)	9 (9-9)	9 (9-9)	9 (9-9)	9 (9-9)	9 (9-9)	**<0.001**
Repeat size (99° percentile)	11	10	11	10	**12**	
Frequency of allele with >50 repeats	0 (0/54784)	0 (0/4774)	0 (0/2612)	0 (0/2296)	0 (0/548)	/
*NUTM2B-AS1*						
Repeat size, median (Q1–Q3)	12 (12–12)	12 (12–12)	12 (9–12)	12 (12–12)	12 (12–13)	**<0.001**
Repeat size (99°/99.9° percentile)	17/21	16/**30**	19/24	18/26	17/**30**	
Frequency of allele with >50 repeats	0.0001 (8/54784)	0.0002 (1/4774)	0.0004 (1/2612)	0 (0/2296)	0 (0/548)	0.229

The box plots highlight the IQR and median. Significant p values with higher values in bold.

* Kruskal-Wallis test/χ2 test, as appropriate.

AFR, Africans; AMR, Americans; EAS, East Asians; EUR, Europeans; SAS, South Asians.

## Discussion

In 2021, Wang *et al*^10^ found the presence of *NOTCH2NLC* CGG expansion in 5/142 (3.5%) individuals with genetically unsolved CMT in a Chinese cohort. Subsequently, CGG expansion in *NOTCH2NLC* was identified in 7/66 (10.6%) undiagnosed CMT cases from Taiwan.^7^ Recent studies have also revealed a high prevalence of CGG expansions in *NOTCH2NLC* (22/1783, 1.2%) and *LRP12* (44/1555, 2.8%), but no case associated with *GIPC1* and *RILPL1*, in Japanese patients diagnosed with CMT2 or dHMN.^8 9^ Notably, in Japan, *LRP12* expansion was reported as the fifth most common cause of CMT after *PMP22*, *MFN2*, *GJB1* and *MPZ*. In these series, the pathological repeat size for *NOTCH2NLC* ranged from 71 to 222, showing no distinct variation from the pathogenic range observed in *NOTCH2NLC*-NIID or *NOTCH2NLC*-OPDM. In contrast, the repeat size for *LRP12* was notably shorter, spanning 50–152 repeat units, compared with that observed in *LRP12*-OPDM.

Sensorimotor neuropathy is now recognised as part of the broader clinical spectrum associated with *NOTCH2NLC* and *LRP12* CGG expansions. Accordingly, some cases diagnosed as CMT may in fact represent phenotypic variants within this continuum, where the disease burden falls predominantly on the peripheral nervous system, rather than a distinct clinical entity. Indeed, Ando *et al*^9^ reported that some CMT patients with *NOTCH2NLC* CGG expansions also exhibited movement disorders and cognitive impairment, which are known features of the *NOTCH2NLC* spectrum. Similarly, Hobara *et al*^8^ described muscular involvement in some patients with *LRP12*-related CMT.

In our study, CGG repeat expansions in *NOTCH2NLC*, *LRP12* and other OPDM-related genes were absent or exceedingly rare from over 560 undiagnosed individuals with CMT, mostly of Northern European ethnicity. This finding is consistent with a previous study^17^ that screened ~20 000 European patients affected by neurological diseases for *NOTCH2NLC* expansion and identified only a single case diagnosed with NIID harbouring a pathogenic expansion.

We believe that the discrepancy between our and previous studies is likely attributable to a real variation in the epidemiology and geographical distribution of diseases associated with CGG expansions in these genes. This was supported by our analysis of over 32 000 ethnically diverse controls, which revealed larger non-pathogenic intermediate alleles in *NOTCH2NLC*, *LRP12, GIPC1*, *NUTM2B-AS1* and *RILPL1* in East Asians (*GIPC1* was also relatively large in Africans), while *ABCD3* repeat size tended to be more expanded in Europeans. Indeed, as repeat expansion disorders are thought to arise from large normal polymorphic repeats, the higher frequency of large non-pathogenic alleles in *NOTCH2NLC* and *LRP12* in East Asians could explain their ancestry-specific propensity to further expand into the fully pathogenic range, and thus the presence in East Asians of *NOTCH2NLC*- and *LRP12*-peripheral neuropathy. Conversely, disorders such as *FXN*-Friedreich’s ataxia,^18^ *C9orf72*-Amyotrophic Lateral Sclerosis/Frontotemporal Dementia,^19^ *FGF14*- Spinocerebellar ataxia 27B^20^ and *RFC1*-cerebellar ataxia, neuropathy, vestibular areflexia syndrome^16^ appear to be relatively more common in Europeans compared with East Asians.

We acknowledge that precise sizing of alleles larger than 50 repeats is limited on srWGS data; therefore, it was not possible to distinguish alleles falling within a ‘grey zone’ of uncertain pathogenicity (meaning expansions >50 repeats but <~70 repeats, which is often the threshold observed for CGG repeats to be toxic)^21^ from those in the full pathogenic range. Also, srWGS was performed on DNA extracted from blood, which may further underestimate the actual repeat size in affected tissues due to the somatic instability of the repeat. Moreover, srWGS, as opposed to the novel long read sequencing techniques, does not provide information on methylation status, which is an important epigenetic factor influencing the expression of the repeat-containing transcript and, consequently, impacting on disease penetrance and expressivity.^22^

Although we acknowledge that the 100K GP is Eurocentric, and further analyses on more heterogeneous and diverse large-scale WGS datasets are warranted to determine the prevalence of CGG expansion in ethnically diverse populations, our study does not suggest that CGG expansions in *NOTCH2NLC* and *LRP12*, or in any of the genes previously associated with OPDM, are a relevant cause of CMT in the UK.

## Supplementary material

10.1136/jnnp-2025-336590 online supplemental file 1

## Data Availability

Full data are available in the Genomic England Secure Research Environment. Access is controlled to protect the privacy and confidentiality of participants in the Genomics England 100K GP and to comply with the consent given by participants for use of their healthcare and genomic data. Access to full data is permitted through the Research Network (https://www.genomicsengland.co.uk/research/academic/join-research-network).
