# Supplementary material for: CGG repeat expansions in Charcot-Marie-Tooth disease: insights from the 100 000 Genomes Project
Source: J Neurol Neurosurg Psychiatry. 2025 Jul 11;97(6):e336590. doi: 10.1136/jnnp-2025-336590 (PMC13217079; doi:10.1136/jnnp-2025-336590)
Supplement: online supplemental file 1 [file jnnp-97-6-s001.docx]

**Supplementary Table 1. Repeat size distribution and frequency of expanded alleles in CMT patients and non-neurological controls.**

|  | **CMT**  **n=560** | **Non-neurological controls**  **n=32,509** | **p value§** |
| --- | --- | --- | --- |
| **Ethnicity** |  |  |  |
| Europeans, n (%) | 495 (88%) | 27392 (84%) | **0.003** |
| South Asians, n (%) | 30 (5%) | 2387 (7%) |  |
| Africans, n (%) | 19 (3%) | 1306 (4%) |  |
| Americans, n (%) | 15 (3%) | 1148 (4%) |  |
| East Asians, n (%) | 1 (<1%) | 276 (<1%) |  |
| 1. ***NOTCH2NLC*** |  |  |  |
| Repeat size, median (Q1-Q3) | 20 (16-22) | 20 (16-22) | 0.331 |
| Repeat size (99° percentile) | 28 | 29 |  |
| Frequency of allele with >50 repeats | 0 (0/1120) | 0.0005 (34/65018) | 0.444 |
| 1. ***LRP12*** |  |  |  |
| Repeat size, median (Q1-Q3) | 11 (10-14) | 11 (10-14) | 0.592 |
| Repeat size (99° percentile) | 16 | 16 |  |
| Frequency of allele with >50 repeats | 0 (0/1120) | 0 (0/65018) | - |
| 1. ***ABCD3*** |  |  |  |
| Repeat size, median (Q1-Q3) | 7 (7-7) | 7 (7-7) | 0.091 |
| Repeat size (99° percentile) | 7 | 7 |  |
| Frequency of allele with >50 repeats | 0 (0/1120) | 0.0002 (11/65018) | 0.663 |
| **D. *GIPC1*** |  |  |  |
| Repeat size, median (Q1-Q3) | 14 (13-15) | 14 (13-15) | 0.751 |
| Repeat size (99° percentile) | 18 | 18 |  |
| Frequency of allele with >50 repeats | 0.009 (1/1120) | 0.0005 (32/65018) | 0.457 |
| **E. *RILPL1*** |  |  |  |
| Repeat size, median (Q1-Q3) | 9 (9-9) | 9 (9-9) | 0.476 |
| Repeat size (99° percentile) | 11 | 11 |  |
| Frequency of allele with >50 repeats | 0 (0/1120) | 0 (0/65018) | - |
| **F. *NUTM2B-AS1*** |  |  |  |
| Repeat size, median (Q1-Q3) | 12 (12-12) | 12 (12-12) | 0.853 |
| Repeat size (99° percentile) | 17 | 17 |  |
| Frequency of allele with >50 repeats | 0 (0/1120) | 0.0002 (10/65018) | 0.678 |

**Legend.** Significant p values with higher values in bold.

**Supplementary Figure 1. Repeat size distribution across non-neurological controls of different ethnic groups.**


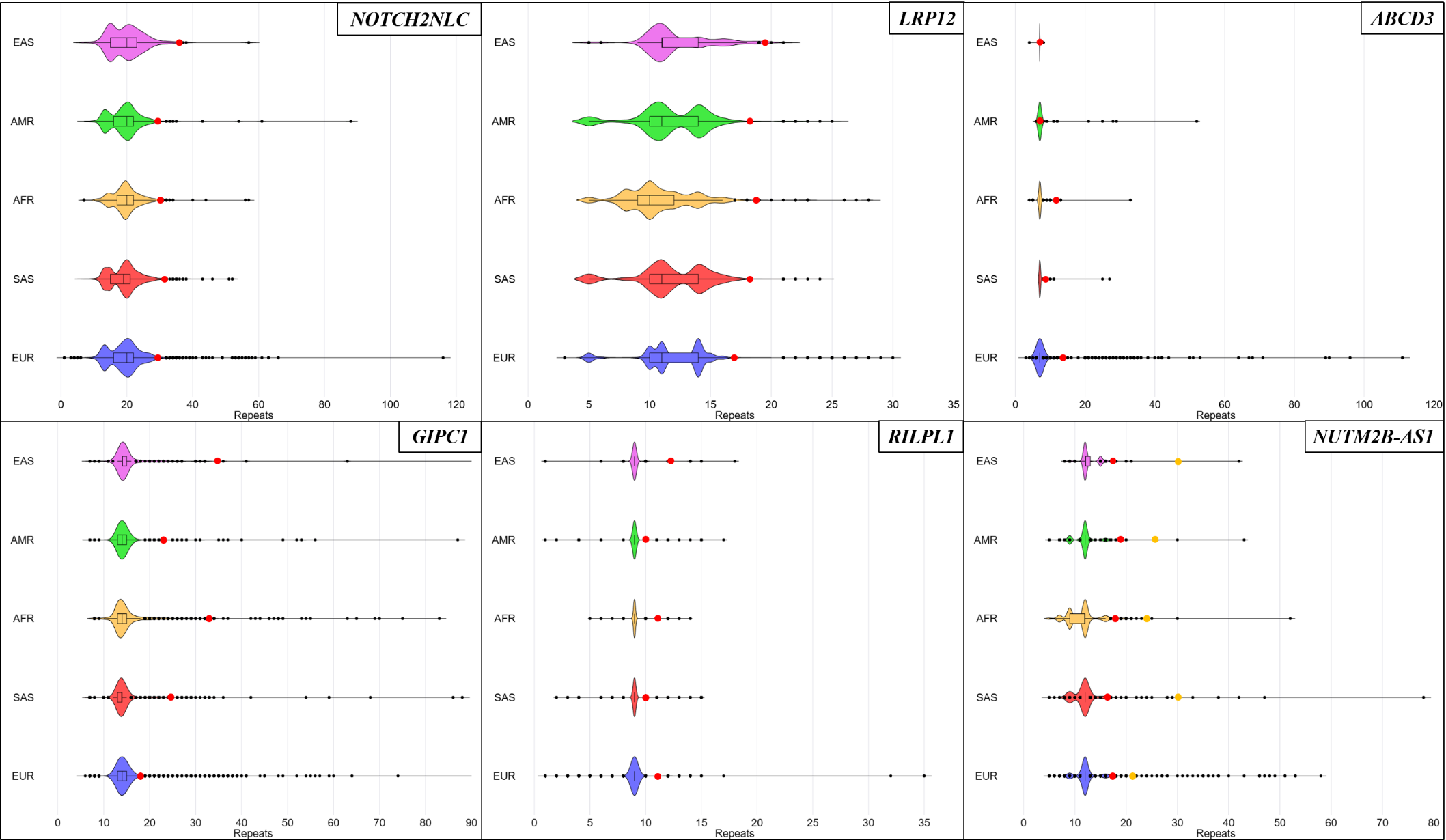


**Legend.** AFR=Africans, n=2612 alleles; AMR=Americans, n=2296 alleles; EAS=East Asians; n=548 alleles; EUR=Europeans, n=54784 alleles; SAS=South Asians, n=4774 alleles. Red dots indicate 99^th^ percentile. Orange dots indicate 99.9^th^ percentile for *NUTM2B-AS1*.
